# The value and safety of percutaneous vertebroplasty (PVP) for spinal compression fractures with the aid of G-arm fluoroscopy

**DOI:** 10.4314/ahs.v25i1.49

**Published:** 2025-03

**Authors:** Guangman Tang, Laiyi Wang, Genyang Jin

**Affiliations:** Department of Orthopedics, The 904^th^ Hospital of the PLA Joint Logistic Support Force, Wuxi, China

**Keywords:** G-arm fluoroscopy, Vertebroplasty, Posterior wall of vertebral body, Vertebral compression fractures, Clinical Application

## Abstract

**Background:**

This study investigated the clinical value and safety of G-arm fluoroscopy-assisted percutaneous vertebroplasty in treating spinal compression fractures with vertebral posterior wall destruction.

**Methods:**

68 patients were divided into a study group (37 patients) and a control group (31 patients). The study group was treated with percutaneous vertebroplasty assisted by G-arm machine, and the control group was treated with percutaneous vertebroplasty assisted by C-arm machine.

**Results:**

At 5 days after operation, there was significant difference between the two groups, and the control group was smaller than the study group. Oswestry disability index (ODI) ODI and Visual analogue scale (VAS) scores of the two groups were significantly lower than those before operation, and ODI and VAS scores of the control group were significantly lower than those of the study group at 5 days after operation. In the control group, bone cement spread to bilateral upper and lower endplates in 21 segments, and bone cement injection volume, which was significantly higher than that in the study group. The operation time of the control group was longer than that of the study group, the above differences were statistically significant (P<0.05).

**Conclusion:**

G-arm fluoroscopy-assisted percutaneous vertebroplasty can effectively relieve pain symptoms, improve postoperative function, and result in minimal surgical trauma with faster recovery.

## Introduction

Trauma, tumors, infections, and other factors are often responsible for vertebral compression fractures. Vertebral compression fractures are often caused by trauma, tumor, infection and other factors. In recent years, the incidence of the disease has increased as China's population ages and related diseaes become more prevalent. With the aging process of China's population and the prevalence of related diseases, the incidence of the disease has increased year by year in recent years^1^. Due to the anatomical and mechanical structure of the thoracolumbar spine, the force borne by the vertebral body is relatively more vertical, and the body weight and external force often produce greater compression load on the thoracolumbar vertebral body, which is more likely to cause the occurrence of spinal compression fractures^2^. Patient's physical examination revealed obvious back pain, tenderness, and percussion pain of injured vertebrae, as well as related symptoms involving nerves. The patient's physical examination showed obvious low back pain, tenderness and percussion pain of injured vertebrae, and even related symptoms involving nerves^3^. In addition, the compliance of conservative treatment of spinal compression fractures is poor, accompanied by complications such as hypostasis pneumonia and lower extremity deep vein thrombosis, which can lead to death in severe cases^4^.

The most common surgical treatment for spinal compression fractures is PVP, which can significantly improve prognosis, reduce pain, and shorten disease progression PVP is the most common surgical treatment for spinal compression fractures, which can significantly improve the prognosis, reduce pain, and shorten the course of disease^5^. Studies have shown that PVP treatment under traditional fluoroscopy has many problems such as multiple fluoroscopy times, pedicle wall perforation, and bone cement leakage. Artificial intelligence technology has led to the application of orthopedic robots in clinical practice. With the rapid development of artificial intelligence technology, orthopedic robot system has been applied in clinical practice^6^. PVP is a minimally invasive spinal surgery for the treatment of spinal compression fractures, which requires accurate injection of bone cement into the vertebral body through pedicle puncture under the guidance of C-arm X-ray machine. Compared with conventional open surgery, PVP has less trauma and faster recovery^7^. However, The pedicle solution is complex, puncture errors and subtle inaccurate operations may increase the risk of iatrogenic puncture injury and leakage of bone cement high surgical accuracy is required, the pedicle solution is complex, puncture errors and subtle inaccurate operations may increase the risk of iatrogenic puncture injury and bone cement leakage. Both doctors and patients are exposed to high-dose radiation, and there may be radiation damage^8^.

C-arm fluoroscopy can only show the perspective image of one direction at a time, so it is necessary to rotate and move the C-arm fluoroscopy frequently during the operation, which greatly prolongs the operation time and significantly increases the number of intraoperative fluoroscopy^9^. For two-segment vertebral fractures requiring one operation, C-arm machine fluoroscopy will have a greater impact on operation times and intraoperative fluoroscopies For two-segment vertebral fractures requiring one operation, the influence of C-arm machine fluoroscopy on operation time and intraoperative fluoroscopy times will be more significant^10^. G-arm X-ray machine (G-arm) is an imaging system superimposed on the C-arm, which can display anteroposterior and lateral images at the same time^11^. G-arm machine fluoroscopy-assisted percutaneous vertebroplasty is more convenient and accurate than C-arm machine in the treatment of spinal compression fractures with vertebral posterior wall destruction, which can effectively shorten the operation time and reduce the number of intraoperative fluoroscopy, thereby reducing the X-ray radiation dose of operators and patients^12^. To investigate the clinical application value and safety of G-arm fluoroscopy-assisted percutaneous vertebroplasty in the treatment of spinal compression fractures with vertebral posterior wall destruction.

## Material and methods

### Research Object

A total of 34 patients with spinal compression fractures with vertebral posterior wall destruction admitted to the Department of Orthopedics of our hospital from December 2020 to December 2022 were included for retrospective analysis. According to the treatment plan, the patients were divided into the study group (37 cases) and the control group (31 cases). All patients were diagnosed with thoracolumbar vertebral compression fractures by imaging evidence and clinical manifestations, and there was no significant difference in baseline indicators between the two groups (P>0.05). See Table 1.

**Table 1:** Comparison of baseline data between the two groups [(x̅ ± s), n (%)]

Baseline Data		Study group (37)	Control group (31)	t	P
Age (years)		68.78±15.35	66.27±14.72	0.254	0.776
Gender n(%)	men	13(35.135)	10(32.258)	0.090	0.956
	women	24(64.865)	21(67.742)		
Causes of fracture n(%)	Trauma	28(75.676)	22(70.968)		
	The tumor	3(8.108)	2(6.452)	2.026	0.917
	infection	2(5.405)	1(2.703)		
	other	4(10.811)	6(16.216)		
	T10	6(3.333)	4(6.667)		
	T11	11(23.333)	9(6.667)		
The vertebral body was involved	T12	13(33.333)	9(6.667)	1.324	0.999
	L1	9(20.000)	9(6.667)		
	L2	4(6.667)	3(6.667)		
	L3	2(6.667)	2(6.667)		
Bone mineral density		-3.51±1.23	-3.48±1.42	0.070	0.933

### Include exclusion criteria

Inclusion criteria: (i) All patients met the diagnostic criteria of thoracolumbar vertebral compression fracture; (ii) All patients had no serious cardiovascular, renal, respiratory diseases, and could tolerate surgery; (iii) Patients have good compliance and can cooperate to complete preoperative index records and postoperative follow-up.

Exclusion criteria: (i) patients with neurological symptoms, super elderly patients or poor tolerance to surgery were excluded; (ii) excluding patients with severe coagulopathy; (iii) excluded patients with poorly controlled spinal tuberculosis.

## Methods

**Figure 1:**
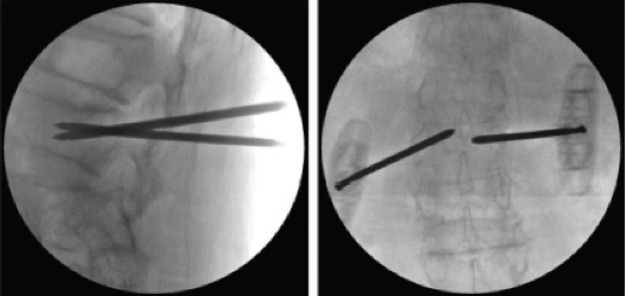
Fluoroscopy-assisted percutaneous vertebroplasty with G-arm machine

The puncture method of the study group was as follow: the patient was placed in the prone position, the chest pad was soft pad, the water belt was placed at the bilateral iliac spine, the abdomen was suspension, the G arm fluoroscopy was used to determine the affected vertebrae and the puncture needle point and mark it, routine skin disinfection, sterile towel, and lidocaine percutaneous local infiltration anesthesia in the direction of the pedicle. Under the fluoroscopic monitoring of the G-arm X-ray machine, the bone puncture needle was inserted into the vertebral body so that the needle tip reached about the anterior one-third of the vertebral body. The bone puncture needle was then removed, the balloon inserted, and the developer injected slowly. The compressed vertebral body was reduced by compression balloon until satisfactory and then injected with toothpast-like bone cement. After waiting for the bone cement to completely fill the expansion space and harden, the puncture needle was pulled out. See Figure 1. After the operation, patients could rest in bed for more than 8 hours, if allowed, they could sit up and ambulate with thoracolumbar fixation brace, and anti-osteoporosis treatment was given at the same time. The puncture method of the control group was bilateral sagittal layered cross puncture, with one needle located at the upper 1/3 of the vertebral body and the other needle located at the lower 1/3 of the vertebral body. Under the guidance of a C-arm X-ray machine (Hitachi DFA200, Hitachi, Japan), the surgical segment was located with Kerner wire, and the pedicle skin entry point was marked for local infiltration and anesthesia. Percutaneous puncture cannula was inserted into the vertebral body under fluoroscopy until the guide needle was located at the front 2/3 of the vertebral body, and a balloon was inserted. The balloon was slowly expanded to reach the ideal position under fluoroscopy monitoring. The balloon was withdrawn and the bone cement was injected continuously at low pressure. The bone cement distribution was monitored by fluoroscopy throughout the injection process until the location distribution was satisfactory. C-arm fluoroscopic positioning, the puncture point and spinous process location were determined and marked according to the locator grid, local infiltration anesthesia was performed, the ideal puncture point and Angle were selected according to the marked trajectory, the puncture needle was placed along the pedicle path until the target puncture location was reached, the pedicle puncture drill was removed, the bone cement was pushed through the cannula, and the injection process was monitored by fluoroscopy. If the bone cement leakage was found, the injection was stopped. After coagulation, the puncture needle was pulled out and sterile dressing was bandaged.

### Observation indicator

**Figure 2:**
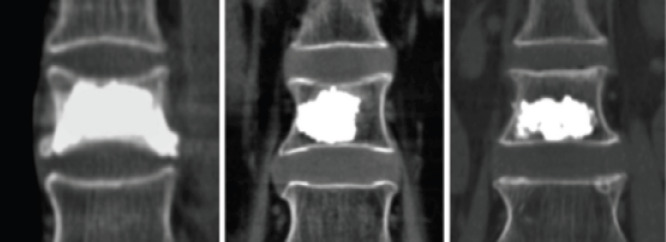
Distribution of bone cement

The operation time, total length of hospital stay, bone cement injection volume, bone cement distribution, VAS score, ODI index, and Cobb Angle before and 5 days after operation were recorded and compared between the two groups. Bone cement distribution: one side of the injection was successful when the bone cement was diffused to the upper and lower endplates. At the same time, the sagittal midline was used to distinguish the two sides of the vertebral body, and the distribution of bone cement was observed. See Figure 2.

**Figure 3:**
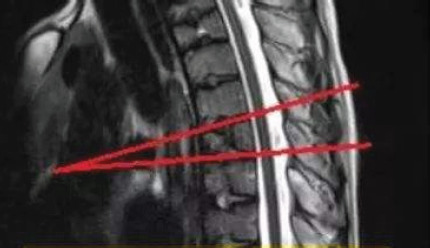
Measurement of Cobb Angle

Visual analogue scale (VAS): 0-10 points, of which 0 points: no pain; Less than 3 points: mild pain, can endure; 4 to 6 points: the patient's pain and sleep are affected, which can be tolerated; 7-10 points: the patient has gradually intense pain, pain is unbearable, affecting appetite and sleep. Oswestry disability index (ODI) was composed of 10 questions, including pain intensity, self-care, standing, sleep, sexual life, etc. The higher the score, the more severe the dysfunction. Cobb Angle measurement: The Cobb Angle of the affected vertebra was measured by the auxiliary tool in the PACS imaging system. See Figure 3. The patients were followed up for 1 year after discharge, and the clinical data at the last follow-up of each group were recorded and compared.

### Statistical analysis

In our study, Excel was used for data sorting, and Statistical Product and Service Solutions (SPSS) 28.0 statistical software (IBM, Armonk, NY, USA) was used for data analysis. According to different data types, different statistical methods are used. The measurement data are expressed by (xTJ+SD). First, the homogeneity of variance test is carried out. If the data are homogenous, the t test is used. Paired t-test, if the variance of the data is unequal, the rank-sum test is used; the count data are all used the chi-square test, and the statistical P<0.05 is significant.

## Results

### Comparison of Cobb angle

**Figure 4:**
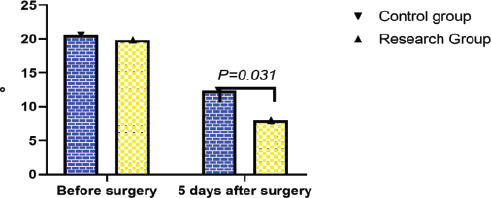
Comparison of Cobb angle (use Epidata to enter daily life self-care ability score comparison data, and then use SPSS 28.0 for statistical processing of the data. The data needs to be entered into a computer database by two people, expressed as mean ± standard deviation, and independent samples are carried out. t test showed that the Cobb angle was significantly reduced in both groups at 5 d after surgery compared to the pre-surgery period (P < 0.05), with the control group being smaller than the study group, and the comparison between the two groups was statistically significant (P < 0.05)

The Cobb angle was significantly reduced in both groups at 5 d after surgery compared to the pre-surgery period (P<0.05), with the control group being smaller than the study group, and the comparison between the two groups was statistically significant (P<0.05). See Figure 4.

### ODI and VAS scores were compared

**Figure 5:**
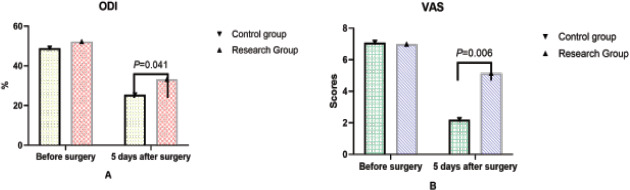
Comparison of ODI and VAS scores (use Epidata to enter daily life self-care ability score comparison data, and then use SPSS 28.0 for statistical processing of the data. The data needs to be entered into a computer database by. And then use SPSS 28.0 for statistical processing of the data. The data needs to be entered into a computer database by two people, expressed as mean ± standard deviation, and independent samples are carried out. t test showed that there were no significant differences in ODI index and VAS score between the two groups before operation (P> 0.05). At 5d after surgery, ODI index and VAS score of the two groups were significantly lower than before surgery, the comparison difference was statistically significant (P< 0.05), and the ODI index and VAS score of the control group were significantly lower than that of the study group at 5d after surgery, the comparison difference was statistically significant (P< 0.05)

There was no significant difference in ODI and VAS scores between the two groups before operation (P > 0.05). At 5 days after operation, the ODI and VAS scores of the two groups were significantly lower than those before operation, and the difference was statistically significant (P < 0.05). The ODI index and VAS score of the control group were significantly lower than those of the study group 5 days after operation, and the differences were statistically significant (P < 0.05). See Figure 5.

### Comparison of related indexes of operation and hospitalization between the two groups

**Figure 6:**
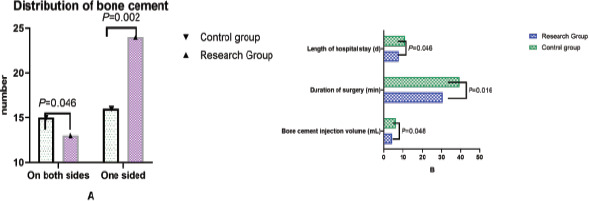
Comparison of related indexes of operation and hospitalization between the two groups (Use Epidata to enter daily life self-care ability score comparison data, and then use SPSS 28.0 for statistical processing of the data. The data needs to be entered into a computer database by two people, expressed as mean ± standard deviation, and independent samples are carried out. t test showed that after surgery, according to the statistical analysis of imaging evidence, bone cement was diffused to the bilateral upper and lower endplates in 21 segments in the control group, which was significantly higher than that in the study group. The bone cement injection volume, hospitalization time, and operation time of the control group were higher than those of the study group, and the differences were statistically significant (P< 0.05)

According to the statistical analysis of imaging evidence after surgery, the bone cement in the control group dispersed to a total of 21 segments of the upper and lower endplates on both sides, which was significantly higher than that in the study group. The bone cement injection amount, hospital stay and operation time in the control group were all higher than that in the study group, with statistical differences (P < 0.05). See Figure 6.

## Discussion

PVP is a new surgical technique, and its mechanism of action is to stabilize the vertebral body by injecting bone cement and other coagulation materials into the diseased vertebral body to prevent further compression and avoid stimulation of peripheral nerves, thus playing a role in pain relief^13^. After injection into the vertebral body, bone cement can restore the biological strength of the diseased vertebral body, enhance the stability of the vertebral body, and its own fixation effect can reduce the fracture stress and reduce the stimulation of the vertebral nerve in the fracture area^14^. The thermal and chemical effects of bone cement polymerization can kill some peripheral nerve cells, thereby reducing pain^15^.

PVP is a minimally invasive surgical method improved on the minimally invasive technique, which has been highly recommended in the treatment of thoracolumbar fractures in recent years. After surgery, it can not only quickly relieve the symptoms of low back pain, but also restore the height of the vertebral body and increase the strength of the injured vertebral body to a certain extent^16^. PVP has the advantages of simple operation, small intraoperative trauma, small blood loss, rapid postoperative pain relief, and small incidence of complications, so it is favored by the majority of clinicians and has been developed and updated for decades^17^. At present, PVP has become the preferred method for the treatment of osteoporotic vertebral compression fractures in clinical practice. At present, this surgical method has been greatly improved and improved compared with the beginning, and localization by imaging means should be relied on before percutaneous operation, which improves the safety factor of such surgery^18^. The working principle of the G-arm X-ray machine is to add an imaging system on the basis of the C-arm X-ray machine, which is equivalent to the vertical superposition of two C-arm X-ray machines^19^. Real-time anteroposterior and lateral images can be obtained without frequent adjustments, which can not only achieve rapid and accurate positioning, reduce the operation time, but also reduce the number of intraoperative fluoroscopy, shorten the exposure time of surgeons and patients under radiation, and reduce the risk of cross infection of patients, which has obvious advantages^20^.

The ODI and VAS scores of the two groups were significantly lower than those before operation. The ODI and VAS scores of the control group were significantly lower than those of the study group at 5 days after operation, and the differences were statistically significant (P < 0.05). The reasons are as follows: because the curved arm used is not a standard circular shape, the C-arm X-ray machine needs to first determine the image in one direction, and then rotate to determine the image in the other direction. It is very difficult to retrieve the initial image, and the position of the machine or the operating table must be readjust, which greatly increases the uncertainty of the position of the surgical target^21^. The new G-arm X-ray machine is a more comprehensive upgrade based on the C-arm X-ray machine, and the equipment is enhanced by X-ray^22^. At the same time, anteroposterior and lateral images can be obtained from two vertical directions, and the position and depth can be judged simultaneously, which greatly increases the accuracy of surgery and reduces the difficulty of operation^23^. In PVP, there is a certain correlation between the volume of bone cement injected into the vertebral body and pain relief. According to relevant studies, the best pain relief effect is achieved when the volume of bone cement injected into the vertebral body reaches 24% of the volume^24^. Under the G-arm X-ray machine, the timing of bone cement injection can be accurately determined, the difficulty of operation can be reduced, and the situation of bone cement injection can be evaluated at any time. Reduce fluoroscopy times and X-ray radiation dose^25^. In PVP under the guidance of traditional C-arm X-ray machine, in order to ensure the safety and smooth implementationf the operation, multiple anteroposterior and lateral fluoroscopy are required during the operation, which increases the number of intraoperative fluoroscopy and the X-ray exposure time of both doctors and patients^26^. Under the guidance of G-arm X-ray machine, anteroposterior and lateral fluoroscopy can be completed in only one fluoroscopy, thus reducing the X-ray exposure time of the surgeon and the patient^27^.

Since the G-arm X-ray machine can simultaneously complete anteroposterior and lateral fluoroscopy imaging, it can effectively avoid the prolongation of operation time caused by different directions of fluoroscopy during PVP under the guidance of traditional C-arm X-ray machine^28^. Because most elderly patients have poor tolerance and are often accompanied by more basic medical diseases, the effect and safety of surgery are greatly affected^29^. If the door can be successfully located at one time during the operation, the direction, Angle and depth of the needle can be monitored in real time, and the position relationship can be determined at any time. During the actual operation, the spinal cord, nerves or other important blood vessels inside and outside the spinal canal can be avoided, so as to improve the safety of the operation^30^.

Our study found that bone cement was diffused to bilateral upper and lower endplates in 21 segments in the control group, which was significantly higher than that in the study group, the bone cement injection volume in the control group was higher than that in the study group, and the operation time in the control group was longer than that in the study group (P < 0.05). The main reasons for this are as follows: the G arm is non-standard round, so it is necessary to determine one direction first and then rotate to determine another direction during fluoroscopy. It is very difficult to retrieve the original image, and the fluoroscopy machine or operating table must be re-adjusted, which is not conducive to the accurate positioning of the surgical target during the operation, and the difficulty of surgical operation will also increase^31^. The G-arm can simultaneously complete the anteroposterior and lateral fluoroscopy in two vertical directions, which is conducive to the judgment of position and depth, accurate intraoperative positioning and reducing the difficulty of surgical operation. It can reduce the risk of intraoperative bone cement leakage and increase the safety of surgical operation^32^. There are uncontrollable factors in the causes of bone cement leakage, and the position of bone cement leakage also has a certain randomness. The most common causes of bone cement leakage are inaccurate puncture position and repeated puncture^5^. In order to prevent cement leakage after surgery, real-time fluoroscopy monitoring and verification are necessary to ensure the safety of surgery. Clear intraoperative images are necessary, which will inevitably increase the risk of cement leakage. High-quality imaging monitoring is considered to be the key to prevent the occurrence of bone cement leakage^33^.

Compared with the C-arm, the G-arm can be used for real-time simultaneous anteroposterior and lateral dual-phase observation, which makes the process of bone cement injection safer. The use of G arm to assist bone cement injection can achieve synchronous forward and lateral close monitoring, which can increase the observation times by 3 to 4 times compared with the conventional C-arm, that is, every 0.5 ml bone cement injection can be observed by fluoroscopy and determine whether there is leakage in the spinal canal and blood vessels, so that the safety of surgery can be guaranteed to agreater extent^34^. However, the price of the G-arm is higher, which may limit its clinical application. The G-arm can simultaneously complete the anteroposterior and lateral fluoroscopy in two vertical directions, which is conducive to the judgment of position and depth, accurate intraoperative positioning and reducing the difficulty of surgical operation^35^. To reduce the risk of intraoperative bone cement leakage and increase the safety of surgical operation. There are uncontrollable factors in the cause of bone cement leakage, and there is a certain randomness in the location of leakage. The most common causes of bone cement leakage are inaccurate puncture position and repeated puncture^36^. Monitoring and verification of real-time fluoroscopy are essential to preventing postoperative cement leakage. In order to prevent postoperative cement leakage, real-time fluoroscopy monitoring and verification are necessary to ensure the safety of the operation. Clear intraoperative images are essential, which will inevitably increase the risk of bone cement leakage, and the leakage may not be clearly observed. High-quality imaging monitoring is considered to be the key to prevent the occurrence of bone cement leakage^37^. Compared with the C-arm, the G-arm can be used for real-time simultaneous anteroposterior and lateral dual-phase observation, which makes the process of bone cement injection safer. Our study has some limitations. The sample size is small, so the number of cases we study is small. Due to the small number of cases, the gender, age, primary disease and specific site of the patient were not further analyzed. In conclusion, percutaneous vertebroplasty assisted by G-arm machine can relieve the pain symptoms of thoracolumbar back and improve the postoperative function of patients. The surgical trauma is small, and the improvement of pain and function is more obvious in the early stage, which can achieve early ambulation.

## References

[1] Chau L, Hu Z, Ko K, Man G, Yeung KH, Law YY (2021). Global sagittal alignment of the spine, pelvis, lower limb after vertebral compression fracture and its effect on quality of life. Bmc Musculoskel Dis.

[2] Kim DH, Vaccaro AR (2006). Osteoporotic compression fractures of the spine; current options and considerations for treatment. Spine J.

[3] Schomig F, Palmowski Y, Nikiforov I, Hartwig T, Pumberger M, Schwabe P (2021). Burst fractures lead to a fracture-associated intervertebral vacuum phenomenon: a case series of 305 traumatic fractures of the thoracolumbar spine. Eur Spine J.

[4] Baranto A, Ekstrom L, Hellstrom M, Lundin O, Holm S, Sward L (2005). Fracture patterns of the adolescent porcine spine: an experimental loading study in bending-compression. Spine.

[5] Buchbinder R, Johnston RV, Rischin KJ, Homik J, Jones CA, Golmohammadi K (2018). Percutaneous vertebroplasty for osteoporotic vertebral compression fracture. Cochrane Db Syst Rev.

[6] Filippiadis DK, Marcia S, Masala S, Deschamps F, Kelekis A (2017). Percutaneous Vertebroplasty and Kyphoplasty: Current Status, New Developments and Old Controversies. Cardiovasc Inter Rad.

[7] Kushchayev SV, Wiener PC, Teytelboym OM, Arrington JA, Khan M, Preul MC (2019). Percutaneous Vertebroplasty: A History of Procedure, Technology, Culture, Specialty, and Economics. Neuroimag Clin N Am.

[8] Wang H, Sribastav SS, Ye F, Yang C, Wang J, Liu H (2015). Comparison of Percutaneous Vertebroplasty and Balloon Kyphoplasty for the Treatment of Single Level Vertebral Compression Fractures: A Meta-analysis of the Literature. Pain Physician.

[9] Zhang H, Xu C, Zhang T, Gao Z, Zhang T (2017). Does Percutaneous Vertebroplasty or Balloon Kyphoplasty for Osteoporotic Vertebral Compression Fractures Increase the Incidence of New Vertebral Fractures? A Meta-Analysis. Pain Physician.

[10] Griffoni C, Lukassen J, Babbi L, Girolami M, Lamartina C, Cecchinato R (2020). Percutaneous vertebroplasty and balloon kyphoplasty in the treatment of osteoporotic vertebral fractures: a prospective randomized comparison. Eur Spine J.

[11] Li Y, Feng X, Pan J, Yang M, Li L, Su Q (2021). Percutaneous Vertebroplasty Versus Kyphoplasty for Thoracolumbar Osteoporotic Vertebral Compression Fractures in Patients with Distant Lumbosacral Pain. Pain Physician.

[12] Rousing R, Kirkegaard AO, Nielsen M, Holtved E, Sorensen LH, Lund T (2020). Percutaneous vertebroplasty as treatment of malignant vertebral lesions: a systematic review and GRADE evaluation resulting in a Danish national clinical guideline. Eur Spine J.

[13] Chien HY, Yang YC, Hsieh MH, Yang CC (2021). Early Percutaneous Vertebroplasty Improves Bone-Cement Integration and Reduces Adjacent Fractures. World Neurosurg.

[14] Xie L, Zhao ZG, Zhang SJ, Hu YB (2017). Percutaneous vertebroplasty versus conservative treatment for osteoporotic vertebral compression fractures: An updated meta-analysis of prospective randomized controlled trials. Int J Surg.

[15] Buchbinder R, Golmohammadi K, Johnston RV, Owen RJ, Homik J, Jones A (2015). Percutaneous vertebroplasty for osteoporotic vertebral compression fracture. Cochrane Db Syst Rev.

[16] Li W, Wang J, Liu W, Xu C, Li W, Zhang K (2021). Machine Learning Applications for the Prediction of Bone Cement Leakage in Percutaneous Vertebroplasty. Front Public Health.

[17] Dai SQ, Qin RQ, Shi X, Yang HL (2021). Percutaneous vertebroplasty versus kyphoplasty for the treatment of neurologically intact osteoporotic Kummell's disease. Bmc Surg.

[18] Yimin Y, Zhiwei R, Wei M, Jha R (2013). Current status of percutaneous vertebroplasty and percutaneous kyphoplasty--a review. Med Sci Monitor.

[19] Lu A, Wang X, Huang D, Feng W (2022). Percutaneous curved vertebroplasty for osteoporotic vertebral compression fracture. Asian J Surg.

[20] Lu W, Wang L, Xie C, Teng Z, Han G, Shi R (2019). Analysis of percutaneous kyphoplasty or short-segmental fixation combined with vertebroplasty in the treatment of Kummell disease. J Orthop Surg Res.

[21] Xie LL, Chen XD, Yang CY, Yan ZL, Zhu J, Quan KQ (2020). Efficacy and complications of (125)I seeds combined with percutaneous vertebroplasty for metastatic spinal tumors: A literature review. Asian J Surg.

[22] Zuo XH, Zhu XP, Bao HG, Xu CJ, Chen H, Gao XZ (2018). Network meta-analysis of percutaneous vertebroplasty, percutaneous kyphoplasty, nerve block, and conservative treatment for nonsurgery options of acute/subacute and chronic osteoporotic vertebral compression fractures (OVCFs) in short-term and long-term effects. Medicine.

[23] Hemama M, El FN, Gana R (2017). Percutaneous vertebroplasty in Moroccan patients with vertebral compression fractures. Pan Afr Med J.

[24] Bonnard E, Foti P, Kastler A, Amoretti N (2017). Percutaneous vertebroplasty under local anaesthesia: feasibility regarding patients' experience. Eur Radiol.

[25] Wu Z, L Y, Mo L, Jiang X, Cui J, Feng Y (2021). Comparison of Cement Leakage Rate and Severity After Percutaneous Vertebroplasty for Osteoporotic Vertebral Compression Fractures Using Front-Opening Versus Side-Opening Cannulas. Orthopedics.

[26] Liang L, Chen X, Jiang W, Li X, Chen J, Wu L (2016). Balloon kyphoplasty or percutaneous vertebroplasty for osteoporotic vertebral compression fracture? An updated systematic review and meta-analysis. Ann Saudi Med.

[27] Lai PJ, Liao JC, Chen LH, Lai PL (2019). Tuberculous spondylitis after percutaneous vertebroplasty: A case series of 9 cases. Biomed J.

[28] Lamy O, Uebelhart B, Aubry-Rozier B (2014). Risks and benefits of percutaneous vertebroplasty or kyphoplasty in the management of osteoporotic vertebral fractures. Osteoporosis Int.

[29] Xu J, Lin J, Li J, Yang Y, Fei Q (2019). “Targeted Percutaneous Vertebroplasty” Versus Traditional Percutaneous Vertebroplasty for Osteoporotic Vertebral Compression Fracture. Surg Innov.

[30] Yu H, Li Y, Yao X, Lin J, Pan Y, Zhuang H (2020). Application of percutaneous vertebroplasty and percutaneous kyphoplasty in treating Kummell's patients with different stages and postural correction status. Expert Rev Med Devic.

[31] Zhao G, Liu X, Li F (2016). Balloon kyphoplasty versus percutaneous vertebroplasty for treatment of osteoporotic vertebral compression fractures (OVCFs). Osteoporosis Int.

[32] Gan DH, Fang MZ, Xue HP, Tan GQ, Li NH, Li ZC (2021). Clinical Observations of Kummell Disease Treatment Through Percutaneous Fixation Combined with Vertebroplasty. Orthop Surg.

[33] Zhang W, Liu S, Liu X, Li X, Wang L, Wan Y (2021). Unilateral percutaneous vertebroplasty for osteoporotic lumbar compression fractures: a comparative study between transverse process root-pedicle approach and conventional transpedicular approach. J Orthop Surg Res.

[34] Ahmadzai H, Campbell S, Archis C, Clark WA (2014). Fat embolism syndrome following percutaneous vertebroplasty: a case report. Spine J.

[35] Li K, Ji C, Luo D, Zhang W, Feng H, Yang K (2021). Role of percutaneous vertebroplasty with high-viscosity cement in the treatment of severe osteoporotic vertebral compression fractures. Sci Rep-Uk.

[36] Denaro L, Longo UG, Papalia R, De Salvatore S, Ruzzini L, Piergentili I (2021). The burden of percutaneous vertebroplasty: an epidemiological nationwide study in Italy from 2009 to 2015. Eur Spine J.

[37] Semaan H, Obri T, Bazerbashi M, Paull D, Liu X, Sarrouj M (2018). Clinical outcome and subsequent sequelae of cement extravasation after percutaneous kyphoplasty and vertebroplasty: a comparative review. Acta Radiol.

